# Ex vivo magnetic resonance imaging of crystalline lens dimensions in chicken

**Published:** 2010-02-02

**Authors:** Rebecca J. Tattersall, Ankush Prashar, Krish D. Singh, Pawel F. Tokarczuk, Jonathan T. Erichsen, Paul M. Hocking, Jeremy A. Guggenheim

**Affiliations:** 1 School of Optometry and Vision Sciences, Cardiff University, Cardiff, UK; 2 Cardiff University Brain and Research Imaging Centre (CUBRIC), School of Psychology, Cardiff University, Cardiff, UK; 3 Experimental Magnetic Resonance Imaging Centre (EMRIC), School of Biosciences, Cardiff University, Cardiff, UK; 4 Scottish Crop Research Institute (SCRI), Dundee, UK; 5 Department of Genetics and Genomics, Roslin Institute and Royal (Dick) School of Veterinary Studies, University of Edinburgh, Roslin, UK

## Abstract

**Purpose:**

A reduction in the power of the crystalline lens during childhood is thought to be important in the emmetropization of the maturing eye. However, in humans and model organisms, little is known about the factors that determine the dimensions of the crystalline lens and in particular whether these different parameters (axial thickness, surface curvatures, equatorial diameter, and volume) are under a common source of control or regulated independently of other aspects of eye size and shape.

**Methods:**

Using chickens from a broiler-layer experimental cross as a model system, three-dimensional magnetic resonance imaging (MRI) scans were obtained at 115-µm isotropic resolution for one eye of 501 individuals aged 3-weeks old. After fixation with paraformaldehyde, the excised eyes were scanned overnight (16 h) in groups of 16 arranged in a 2×2×4 array. Lens dimensions were calculated from each image by fitting a three-dimensional mesh model to the lens, using the semi-automated analysis program mri3dX. The lens dimensions were compared to measures of eye and body size obtained in vivo using techniques that included keratometry and A-scan ultrasonography.

**Results:**

A striking finding was that axial lens thickness measured using ex vivo MRI was only weakly correlated with lens thickness measured in vivo by ultrasonography (r=0.19, p<0.001). In addition, the MRI lens thickness estimates had a lower mean value and much higher variance. Indeed, about one-third of crystalline lenses showed a kidney-shaped appearance instead of the typical biconvex shape. Since repeat MRI scans of the same eye showed a high degree of reproducibility for the scanning and mri3dX analysis steps (the correlation in repeat lens thickness measurements was r=0.95, p<0.001) and a recent report has shown that paraformaldehyde fixation induces a loss of water from the human crystalline lens, it is likely that the tissue fixation step caused a variable degree of shrinkage and a change in shape to the lenses examined here. Despite this serious source of imprecision, we found significant correlations between lens volume and eye/body size (p<0.001) and between lens equatorial diameter and eye/body size (p<0.001) in these chickens.

**Conclusions:**

Our results suggest that certain aspects of lens size (specifically, lens volume and equatorial diameter) are controlled by factors that also regulate the size of the eye and body (presumably, predominantly genetic factors). However, since it has been shown previously that axial lens thickness is regulated almost independently of eye and body size, these results suggest that different systems might operate to control lens volume/diameter and lens thickness in normal chickens.

## Introduction

The crystalline lens is an important determinant of the eye’s refractive state. Several parameters influence lens power: its anterior and posterior surface radii of curvature, its thickness, and its refractive index distribution. Recent studies suggest that, during childhood, the power of the crystalline lens reduces by approximately 2 diopters (D) and that this change offsets most of the myopia that would otherwise be produced by the axial elongation of the maturing eye [1-4]. Despite this important role in normal ocular development, the crystalline lens has received relatively little attention in the emmetropization literature over recent years, probably because visually regulated compensation to imposed blur in animal models seems to occur via changes to tissues other than the crystalline lens [5].

However, an additional reason why most emmetropization studies measure lens thickness but not lens equatorial diameter or surface curvature is that these latter parameters are much more difficult to quantify (in contrast, lens thickness can be measured by ultrasonography or partial coherence interferometry, which are standard procedures in this field of research). Two specialized techniques have been developed to measure the radius of curvature of the anterior and posterior surfaces of the lens in vivo: phakometry and Scheimpflug imaging. In phakometry, one or more point light sources are presented at known positions in front of the eye and the reflections produced by the cornea (Purkinje images I and II) and the crystalline lens (Purkinje images III and IV) are photographed or imaged. The relative sizes and/or positions of the Purkinje images enable the curvatures of the anterior and posterior surface of the crystalline lens to be calculated by ray tracing, similar to the principle used in keratometry. Scheimpflug imaging/photography operates analogously, except that the point source used in phakometry is replaced by a slit source placed with its long axis aligned with the pupil center. Phakometry and Scheimpflug imaging cannot be used to measure the equatorial diameter or volume of the lens.

In studies of emmetropization in animal models, two ex vivo techniques have also been used to obtain detailed measurements of lens size and shape: hemisectioning and frozen sectioning. In hemisectioning, a single cut is made through the approximate center of a freshly excised eye, typically along the sagittal plane (the vertical plane passing through the optical axis that divides the eye into symmetric left and right halves). The hemisected eye is then photographed or imaged alongside a scale bar to enable the axial thickness, anterior and posterior surface radii of curvature, and equatorial diameter of the lens to be determined by image analysis. The two most serious difficulties inherent to the hemisectioning technique are (1) ensuring that the eye is cut exactly through its center, and (2) preventing distortion of the tissue by the mechanical force of the cutting action. Frozen sectioning is a related technique in which the excised eye is mounted on a freezing microtome and then photographed or imaged after 10–30-µm-thick sections are serially removed from it. By mounting the eye with its optical axis parallel to the stage of the freezing microtome, the removal of sections provides views of the crystalline lens analogous to those produced by hemisectioning. However, advantages of frozen sectioning over hemisectioning are that (1) less tissue distortion occurs because the eye gains mechanical strength by being frozen and because only thin sections are removed from its surface, (2) several images can be examined to identify the one corresponding approximately to the optical axis of the eye—usually this is deemed to be the image in which lens thickness is maximal, and (3) it has the potential to measure lens volume if the eye is photographed/imaged after every section is removed and the thickness of the sections is known.

To our knowledge the only other technique that is able to provide estimates of all of the key structural parameters of the lens (i.e., axial thickness, equatorial diameter, surface curvatures, and volume) is magnetic resonance imaging (MRI) scanning. Being a noninvasive technique, MRI can be used for both in vivo and ex vivo samples. An advantage of MRI scanning over frozen sectioning is that multiple ex vivo tissue samples (or for in vivo analysis of small animals, multiple anesthetized animals) can be scanned simultaneously, thereby greatly increasing throughput. Because of these advantages, we chose to use MRI to quantify crystalline lens parameters in a group of normal chickens that had been phenotyped in order to (1) explore correlations between the growth of different parts of the eye and between the growth of the eye and the body as a whole, and (2) map quantitative trait loci controlling specific ocular component dimensions [6].

## Methods

### In vivo phenotypic assessment

The experimental procedures involving animals complied with UK Home Office regulations and were in compliance with the ARVO Statement for the Use of Animals in Ophthalmic and Visual Research. Details of the in vivo phenotypic assessment procedures have been described previously [6]. Briefly, chickens from the F_10_ generation of a broiler-layer advanced intercross line [6] were hatched in groups (“hatches”) of about 20 chicks per week and raised under uniform environmental conditions (12 h:12 h light–dark cycle). Illumination in the brooders was 250–300 lux. At age 3 weeks, chickens were weighed, anesthetized with an intramuscular injection of ketamine and xylazine (75 mg and 5 mg, respectively, per kg body weight) and examined using video-keratometry and high-resolution A-scan ultrasonography to obtain data on corneal curvature and axial ocular component dimensions (corneal thickness, anterior chamber depth, lens thickness, and vitreous chamber depth). After an overdose of anesthetic (an intraperitoneal injection of approximately 100 mg sodium pentobarbital) body length was measured from the beak to hock, and the eyes were enucleated. Extraneous orbital tissues, such as muscle and conjunctiva, were removed using fine scissors under a dissection microscope. Equatorial eye diameter was measured with a calibrated video camera system. Eyes were weighed on a digital balance, placed in about 10 ml of freshly prepared 4% paraformaldehyde (extra pure grade, Sigma Chemical Company, Poole, UK) in phosphate-buffered avian saline (10 mM sodium phosphate buffer, 128 mM NaCl, pH 7.2) and stored at 4 °C for 6–12 months in readiness for MRI. Sex was determined using a PCR-based assay, with DNA extracted from a 2 ml blood sample (collected by cardiac puncture, using EDTA as an anticoagulant) [7].

### Magnetic resonance imaging

Due to the high correlation in eye size parameters between fellow eyes, it was decided to scan only one eye of each chicken. Furthermore, we chose to carry out the MRI scans on ex vivo eyes because (1) this enabled multiple eyes to be scanned simultaneously, which increased throughput and reduced costs, and (2) it permitted longer scan times than would have been possible with anesthetized animals, which facilitated imaging at high resolution with a high signal-to-noise ratio. All MRI scans were performed on left eyes, except where the left eye was unavailable as a result of damage during enucleation or trimming (n=2 eyes), in which case the right eye was scanned.

**Figure 1:**
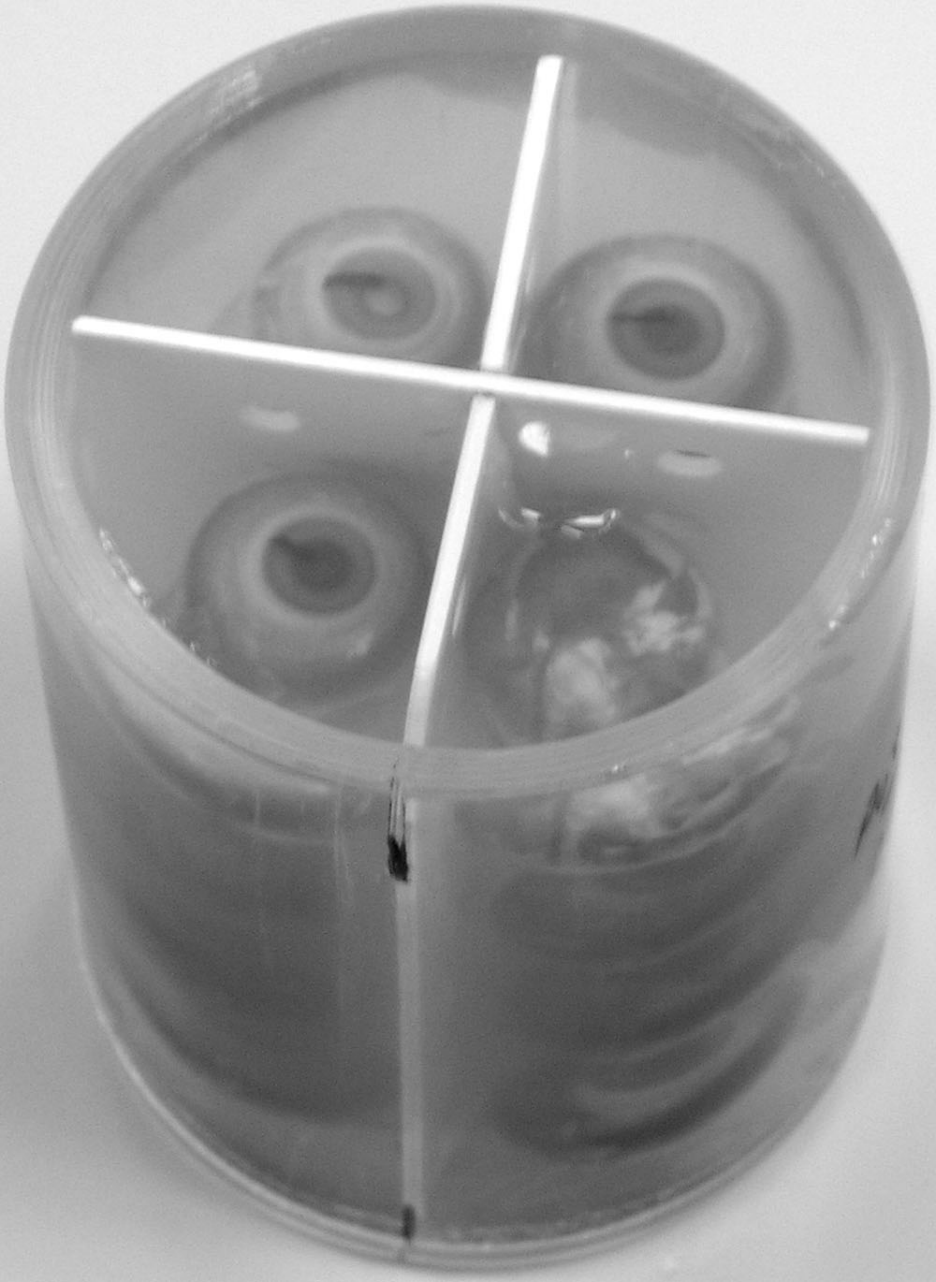
Arrangement of eyes for magnetic resonance imaging scanning. The figure shows 16 paraformaldehyde-fixed chicken eyes embedded in low-melting-point agarose in a 2×2×4 array in readiness for an overnight magnetic resonance imaging (MRI) scan. Note that the eye in the lower-right quadrant of the uppermost layer was positioned in an inverted orientation to permit unambiguous identification of each eye in the resultant MRI image. In the remaining three eyes of the uppermost layer, it is possible to see the ink mark on the nasal cornea, which was used to indicate the original orientation of the eye in the head.

Eyes were scanned in groups of 16 at a time. Four eyes were removed from their fixative solution and arranged in a single 2×2 layer inside a solid-based Perspex cylinder (Amari Plastics Ltd, Cardiff, UK) of internal diameter 38 mm and external diameter 43 mm. The eyes were maintained in a defined position as a layer of molten (37 °C), 1%, low-melting-point agarose (Product BPE165-25; Fisher Scientific Ltd, Loughborough, UK) solution was allowed to solidify around them. The next four eyes were arranged above the first four, and their positions fixed using a further layer of molten agarose solution. This process was repeated twice more until a four-layer arrangement of the eyes was achieved. All eyes were embedded in the same orientation (which could be inferred from an ink mark on the nasal cornea and the position of the optic nerve), except for the last of the 16 eyes, which was placed in an inverted orientation to allow unambiguous identification of each eye in the scanned images (Figure 1). Once the last layer of agarose had solidified, the Perspex cylinder was covered with laboratory film and stored at 4 °C until scanned later the same evening.

For MRI scanning, the Perspex cylinder was placed with its long axis parallel to the long axis of a Bruker Biospin Avance 9.4 Tesla, 20-cm diameter bore MRI scanner (Bruker Ltd, Karlsruhe, Germany). A quadrature birdcage-style coil of internal diameter 72 mm was used as a single transmission/receiver coil. The cylinder was scanned for 16 h using a (mostly) T_2_-weighted TURBO-RARE three-dimensional (3D) sequence with a 512×384×384 voxel array at 115-μm isotropic resolution. The scan parameters were: Echo spacing (∆TE)=25 ms, RARE factor (ETL)=4, Effective echo time (TE_eff_)=50 ms, Repetition time (TR)=775 ms, Read-out bandwidth (BW)=138.9 kHz, and Sampling dwell (DW)=7.2 μs. The raw image files were loaded into the ImageJ program [8], and each eye was sequentially “cropped” out and saved as an individual file in Analyze® format using ImageJ. The image was smoothed using a Gaussian function (kernel size 0.1mm) and loaded into the mri3dX analysis program [9]. The crystalline lens was flood filled, using a thresholding algorithm, and then each “slice” of the image was manually checked and, if necessary, unfilled regions or edges of the lens were manually filled. Next a virtual mesh construct of 32,768 triangular polygons was shrink wrapped in three dimensions over the flood-filled lens [9]. The vertices of the mesh model were then smoothed to remove surface undulations inherent to the polygonal mesh. From this final virtual 3D model, the axial thickness, equatorial diameter, volume, and surface curvatures of the lens were calculated. Axial thickness was measured along a line running from a user-defined point specifying the center of the anterior surface of the lens that passed through the geometric center of the lens (approximating the optical axis). Equatorial diameter was estimated by finding the maximum width of the lens mesh model in a plane orthogonal to the approximated optical axis. Surface curvatures were estimated by finding the best-fit curve for the 3D surface of the lens over an area that subtended 60° to the “optical axis” line, using Powell’s algorithm [10].

The order in which the 501 eyes were scanned was randomized (such that eyes from chickens that were hatched and phenotyped together were generally MRI scanned in different sessions). After MRI scanning, all eyes were carefully removed from their agarose-embedding medium, cleaned of residual agarose using forceps, and returned to their original container of 4% paraformaldehyde at 4 °C so that the operator was blind as to whether or not an eye had been scanned previously. The eyes of 19 randomly selected chickens were scanned a second time to permit an evaluation of the reproducibility of the scanning and mri3dX analysis routines.

### Statistical analysis

All statistical analyses were performed using SPSS version 12 (SPSS Inc., Chicago, IL). The normality of traits was assessed using the Kolmogorov–Smirnov test. Correlations between traits were assessed using the Spearman rank correlation test because this test is valid for nonnormal trait distributions. Binary logistic regression was used to test whether the categorical variables “hatch” (i.e., chickens hatched and phenotyped together) and “scan group” (i.e., chickens whose eyes were MRI scanned together) were associated with lenses being rated as either kidney-shaped or biconvex. A p value of <0.05 was taken to indicate statistical significance.

## Results

### Scanning parameters and kidney-shaped lenses

MRI scanning and analysis were performed on 501 eyes of 501 3-week-old chickens. We decided that scans would be conducted overnight (16 h duration) since this provided a good compromise between the number of eyes that could be scanned simultaneously and the resulting image resolution and contrast. Preliminary trials showed that 16 eyes could be scanned simultaneously at moderate resolution (resulting image voxel size=115 μm in each dimension), yet providing sufficiently high contrast for semi-automated image analysis.

**Figure 2:**
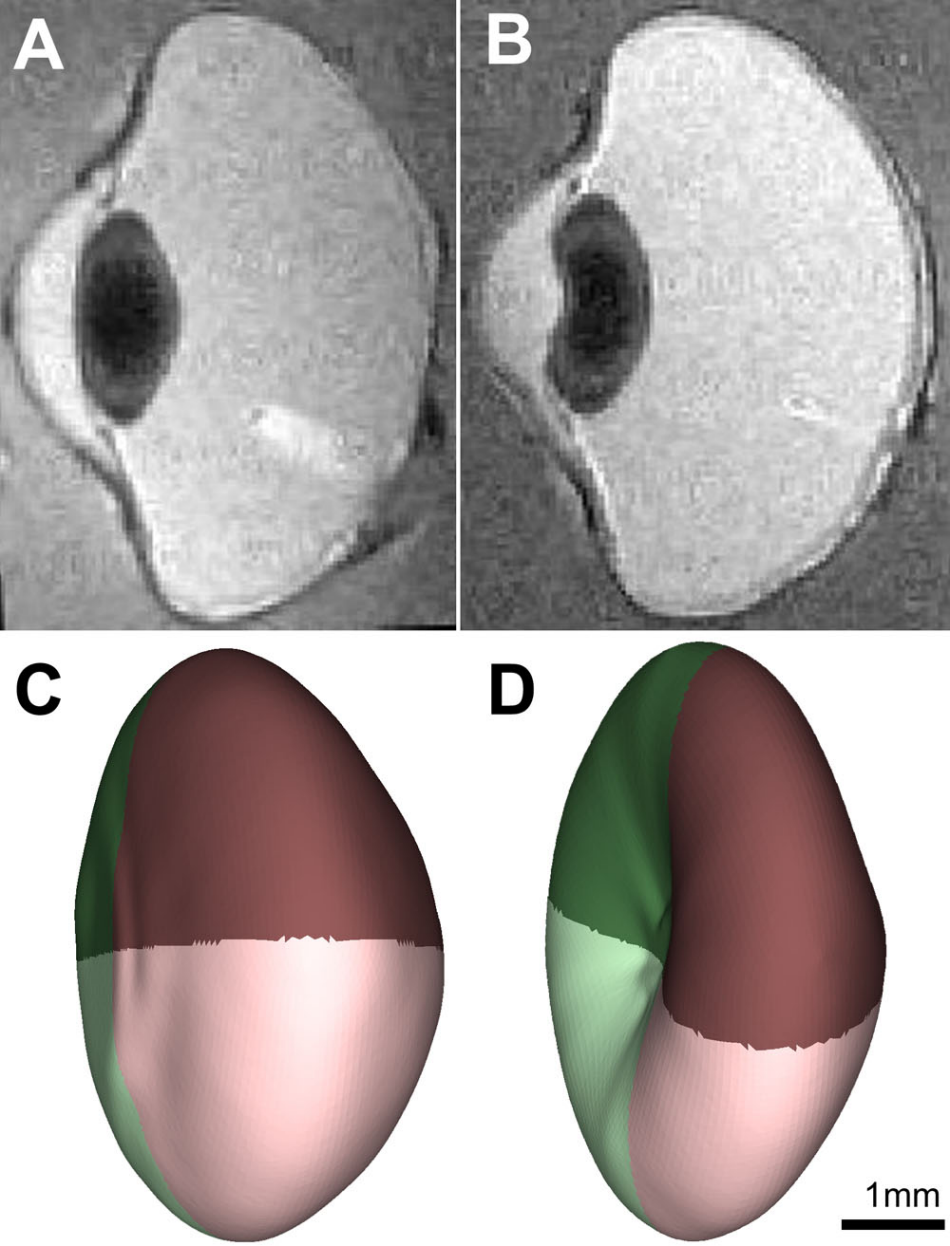
Typical appearance of kidney-shaped and biconvex  lenses. Panels **A** and **B** each show a single “slice” from the central region of a magnetic resonance imaging (MRI) scan: a nonkidney- shaped lens (**A**) and a kidney-shaped lens (**B**). Panels **C** and **D** show MRI3dX mesh models of the lenses from two eyes: a lens with a normal biconvex appearance (**C**) and a lens showing a depression the anterior surface, characteristic of kidney-shaped lenses (**D**). The scale bar in panel **D** is only an approximation since the true dimensions are altered due to the presentation in perspective.

**Figure 3:**
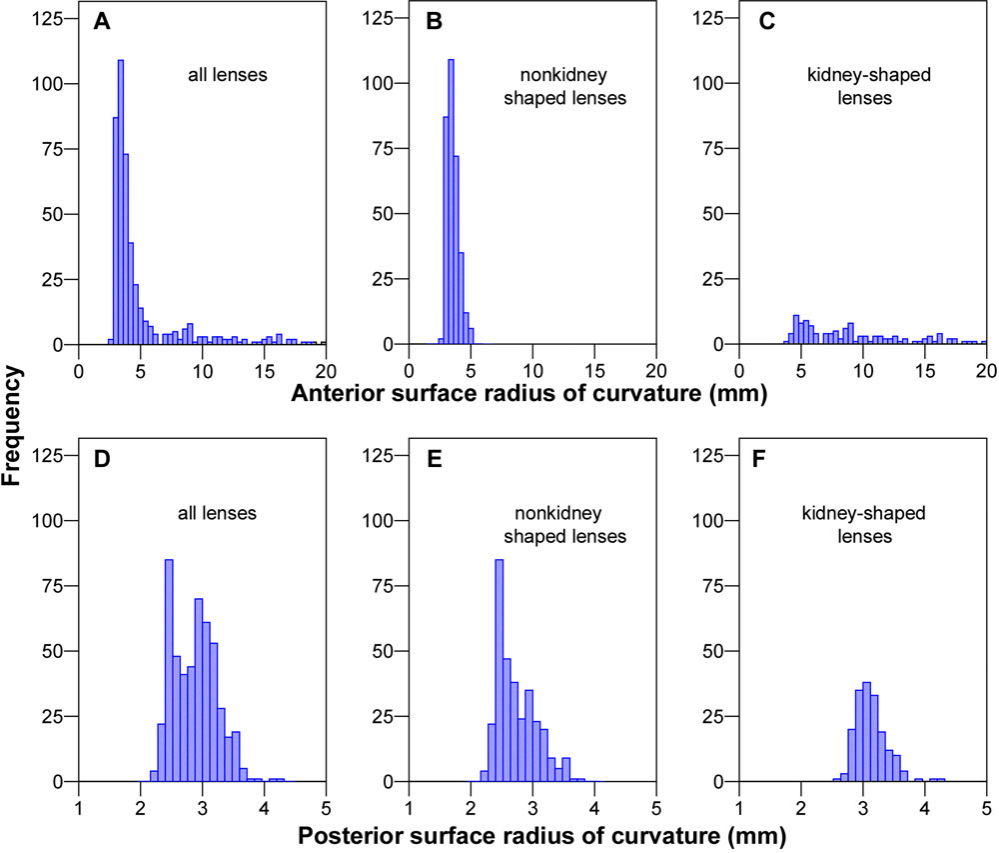
Frequency distributions of crystalline lens surface curvatures obtained using magnetic resonance imaging. Panels **A**-**C** show data for the anterior surface radius of curvature. Panels **D**-**F** show data for the posterior surface radius of curvature. Panels **A** and **D** show data for all eyes. Panels **B** and **E** show data for eyes judged subjectively to be nonkidney shaped. Panels **C** and **F** show data for eyes judged to be kidney shaped. Note that the *x*-axis scale for panel **A** has been truncated to aid visualization. It is apparent that the posterior surface radius is more normally distributed than the anterior surface (the latter shows a greater degree of skew).

Surprisingly, even though the lenses of most eyes had a typical biconvex shape (Figure 2A,C), approximately one-third of the lenses had an obvious kidney shape, characterized by a concave depression in their anterior surface (Figure 2B,D). Histogram plots of the anterior and posterior surface radii of curvature suggested that the anterior surface was more varied in its range of curvature (Figure 3A,C) than was the posterior surface (Figure 3D-F) even in eyes that did not have a kidney-shaped appearance. However, neither surface showed a normal frequency distribution of radii values (unlike the in vivo ocular traits measured previously in these chickens [6]). When lenses subjectively rated as kidney shaped (Figure 3C,F) were removed, the skew in the anterior surface radius frequency distribution of the nonkidney-shaped lenses was much diminished but both the anterior and posterior distributions remained nonnormal (Figure 3B,E; Kolmogorov–Smirnov test, p<0.005). On the assumption that the kidney-shape effect was an artifact of the experimental protocol rather than a natural variation (see below), this suggested that the factor or factors leading to the artifact did not act in an all-or-nothing manner and that a clear objective method of separating lenses into affected and unaffected categories was not possible.

### Reproducibility of repeat scans of the same eye

A random sample of 19 eyes was scanned twice to evaluate the reproducibility of the scanning and mri3dX analysis routines. For this set of 19 eyes, there were no significant differences in the various measurements obtained when the lenses were scanned first compared to when they were scanned a second time (paired *t* test, p=0.14–0.60). The correlations between the first and second sets of measurements were generally high (Table 1), with the most reproducibly measured trait being axial lens thickness (r=0.92, p<0.001). However, lens equatorial diameter showed a much lower level of correlation between repeat measurements (r=0.38, p=0.11). Similar results were found for nonkidney-shaped lenses only (Table 1). These results suggested that the combination of MRI scanning and mri3dX image analysis was unlikely to have been the cause of the kidney-shape artifact. Furthermore, they suggested that even though the scanning and analysis were subject to measurement “noise,” intersubject variation of trait dimensions could still be reliably distinguished for most traits, using our MRI analysis method.

**Table 1:** Correlation of lens dimensions between two MRI scans.

**Group**	**Axial lens thickness**	**Lens equatorial diameter**	**Lens volume**	**Anterior surface curvature**	**Posterior surface curvature**
All lenses (n=19)	0.915 (p<0.001)	0.381 (p=0.108)	0.653 (p=0.002)	0.809 (p<0.001)	0.865 (p<0.001)
Non kidney-shaped lenses only (n=15)	0.838 (p<0.001)	0.259 (p=0.351)	0.714 (p=0.003)	0.610 (p=0.016)	0.821 (p<0.001)

### Comparison between in vivo ultrasound and ex vivo magnetic resonance imaging measurements

**Figure 4:**
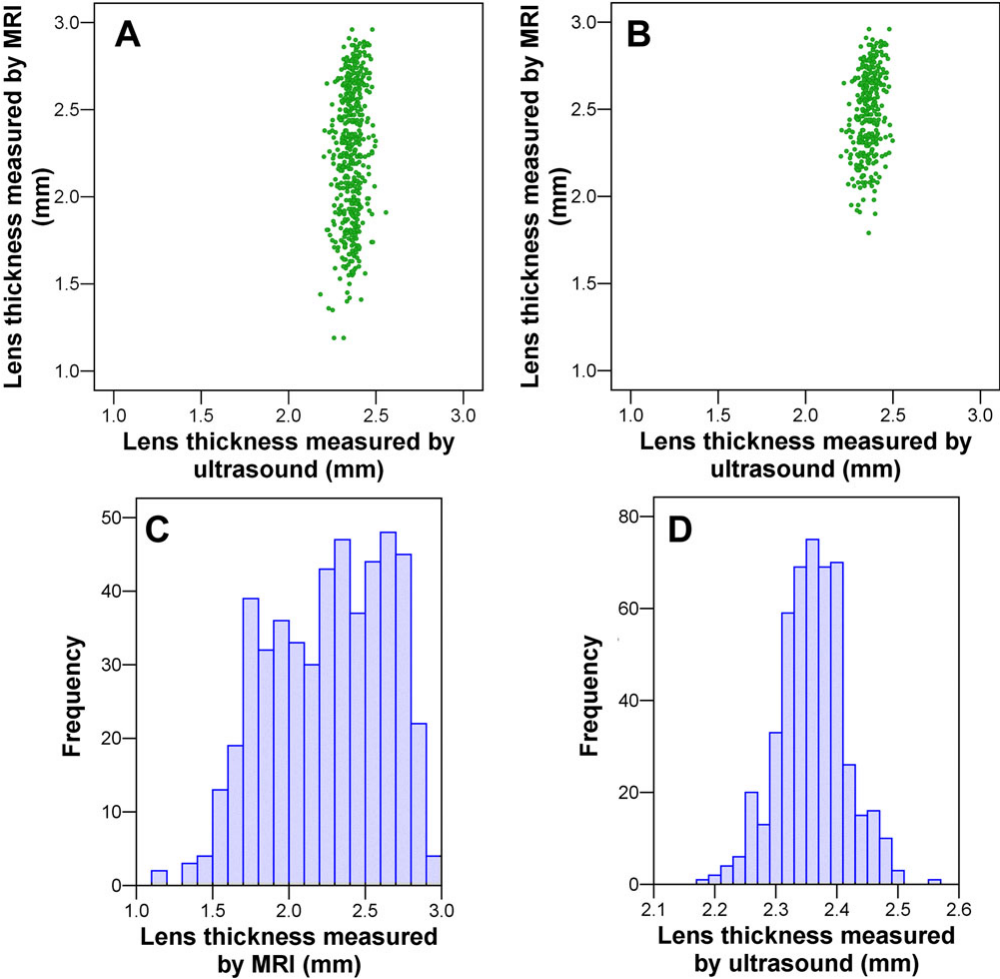
Comparison of axial lens thickness measured using in vivo A-scan ultrasonography and ex vivo magnetic resonance imaging. Panels **A** and **B** show scatter plots of axial lens thickness measured using the two techniques, for all lenses (**A**) and lenses judged subjectively to be nonkidney shaped only (**B**). Panels **C** and **D** show frequency distribution histograms of axial lens thickness for all lenses measured using magnetic resonance imaging (MRI) (**C**) and ultrasound (**D**).

Data were available for axial lens thickness from both in vivo A-scan ultrasonography and ex vivo MRI analysis (n=492 and n=501 eyes, respectively). The Spearman correlation between the two measures of lens thickness was r=0.19 (n=492, p<0.001) for all lenses and r=0.30 (n=316, p<0.001) for those lenses subjectively rated as nonkidney shaped. Scatter plots of this relationship are shown in Figure 4 (Figure 4A, all lenses; Figure 4B, lenses judged to be nonkidney shaped). The frequency distribution of axial lens thickness measured using MRI had a broad positively skewed distribution centered at about 2.25 mm (Figure 4C). In contrast, axial lens thickness measured in vivo using ultrasound had a narrow normal distribution centered at about 2.35 mm (Figure 4D). Thus, even though the repeat-scan analysis showed that axial lens thickness was the most reproducibly measured trait using MRI, the ex vivo results appeared to be subject to a source of measurement error, most likely related to the kidney-shape-inducing artifact. Moreover, restricting the analysis to those lenses that were subjectively “normal” in appearance (by excluding kidney-shaped lenses) did little to remove the influence of the shape-inducing artifact.

To explore this idea further, we examined the correlations between axial lens thickness and all of the other ocular and nonocular traits that were measured, first using the data for lens thickness measured by MRI and then using the data for lens thickness measured by ultrasonography (Appendix 1). Irrespective of whether all eyes or just eyes with nonkidney-shaped lenses were considered, the correlations between lens thickness and other lens parameters were much higher when lens thickness was measured by MRI than by ultrasound, suggesting that the MRI lens–lens trait correlations were biased upward (Appendix 1). This might have occurred if several crystalline lens traits were influenced together by the shrinkage artifact. For example, if both the axial thickness and the anterior surface curvature of the lens varied as a function of the degree of shrinkage, then these two traits might become correlated to one another for lenses in the post-shrinkage state, even if they happened to be uncorrelated initially. For the comparisons between lens thickness and nonlens trait dimensions, however, the correlations appeared more reliable; that is, correlations—and particularly their significance levels—were more similar for the two methods of measuring lens thickness, especially for the lenses judged to be nonkidney shaped (Appendix 1).

Thus, despite the adverse effects of the artifact phenomenon, the high statistical significance of the in vivo versus ex vivo comparison of axial lens thickness values suggested that the MRI data could still be used to explore the statistical significance—if not, perhaps, the magnitude—of correlations between lens parameters and nonlens traits (comparisons that have rarely been possible in the past due to the difficulty of measuring the size and shape of the crystalline lens, either in vivo or ex vivo).

### Correlations between traits

**Figure 5:**
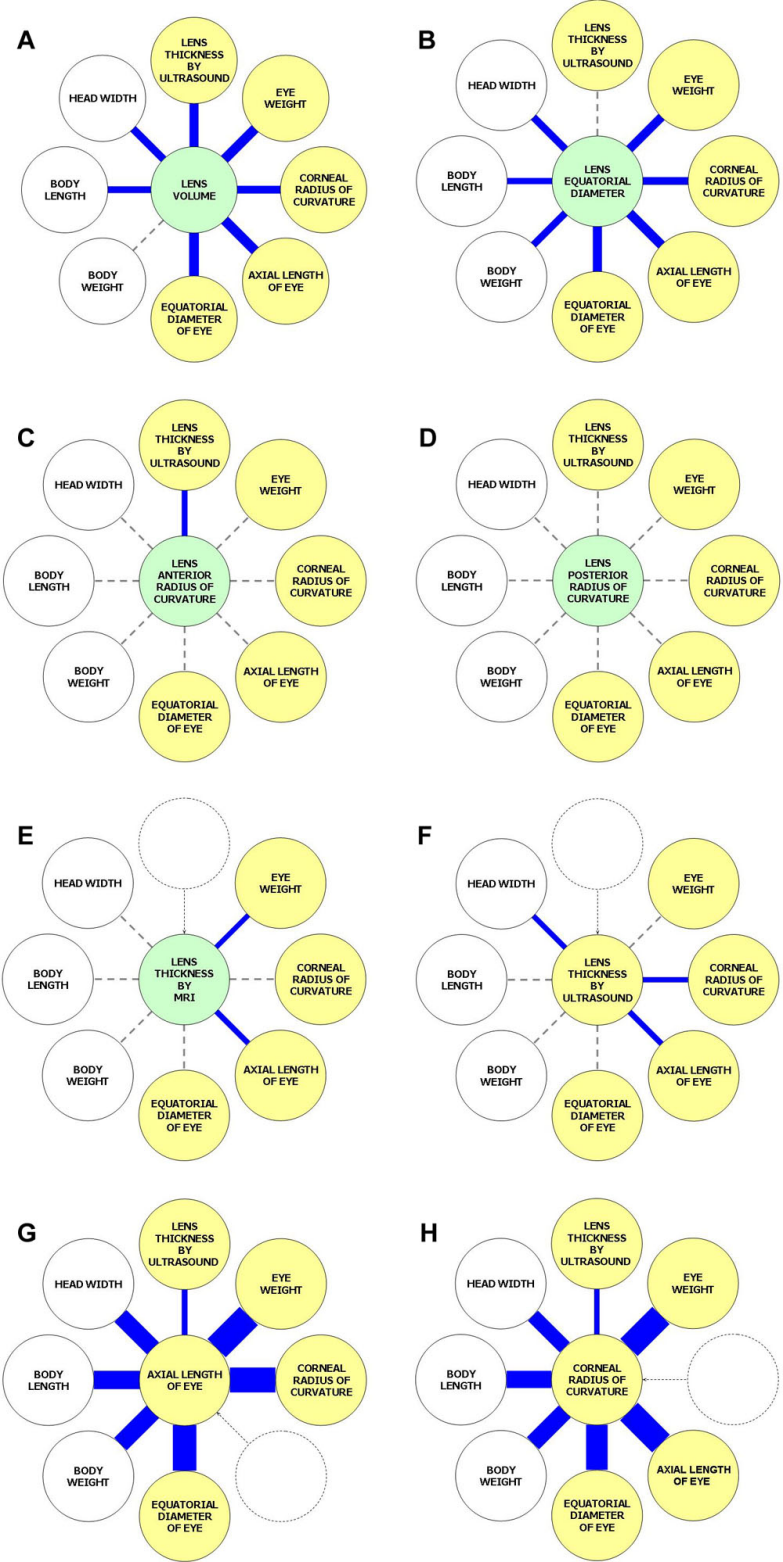
Illustration of significant correlations between lens, eye and body size traits (for eyes with lenses judged as non-kidney shaped). Panels **A**-**H** each depict the degree of correlation between an individual trait (central circle) and a range of other eye and body size traits (outer circles). Significant correlations between traits are indicated by blue lines, with thickness proportional to the magnitude of the correlation coefficient. Results are shown for lens volume (**A**), lens equatorial diameter (**B**), lens anterior radius of curvature (**C**), lens posterior radius of curvature (**D**), axial lens thickness measured by magnetic resonance imaging (MRI) (**E**), axial lens thickness measured by ultrasonography (**F**), axial eye length (**G**), and corneal radius of curvature (**H**). Ocular traits measured by MRI are depicted as green circles, ocular traits measured by methods other than MRI as yellow circles, and nonocular traits as white circles. The data for panels **G** and **H** have been published previously [6] and are included here to provide a comparison between lens traits and other ocular traits.

Correlations between the various lens parameters measured and various other ocular and nonocular traits were calculated, first for all of the chickens examined (n=501) and second for those chickens whose lenses were subjectively rated as having a nonkidney shape (n=323). The results are shown in full in Appendix 2, and the major findings are illustrated in Figure 5. Lens volume (Figure 5A) and lens equatorial diameter (Figure 5B) were highly correlated (p≤0.001) with all of the other ocular traits and with most body size traits. In marked contrast lens anterior and posterior radii of curvature were unrelated to all of the other nonlens traits (Figure 5C,D). As reported previously [6] axial lens thickness was intermediate, being significantly correlated with a limited number of other (nonlens) ocular and body size traits (Figure 5E,F). For comparison, Figure 5 also depicts analogous results for axial eye length (Figure 5G) and corneal radius of curvature (Figure 5H) measured using ultrasonography and video-keratometry, respectively, in the same chickens [6]. For these nonlens traits, the correlations between the various eye size traits and between eye and body size traits were uniformly high. Thus, the general pattern was that lens volume and lens equatorial diameter were more closely correlated with eye and body size than were axial lens thickness and the lens surface curvatures but that the size of the crystalline lens was not tightly related to overall eye size or body size in comparison to traits such as axial length.

## Discussion

### Measurement of lens dimensions

Only a limited number of methods have been developed to measure the dimensions of the crystalline lens. In vivo optical methods, such as phakometry and Scheimpflug imaging, have proven successful in determining the anterior and posterior surface curvatures of the lens and the axial lens thickness. Following pupil dilation these techniques allow a wide-angle view of the anterior surface of the lens, along with a more restricted view of the posterior surface. Ray-tracing algorithms must be used to compensate for the effect of refraction at the anterior and posterior surfaces of the cornea and, in the case of estimation of the position and curvature of the posterior surface of the lens, refraction at the anterior surface and through the lens itself (the latter analysis being complicated by the gradient index of the lens). However, phakometry and Scheimpflug imaging do not permit estimation of lens equatorial diameter or lens volume. To our knowledge, these techniques have not been used to study avian eyes.

Hemisectioning and frozen sectioning allow a more complete assessment of the size and shape of the lens than do in vivo phakometry and Scheimpflug imaging. Both sectioning techniques have been used successfully in studies of the chicken eye [11-13]. Of the two approaches frozen sectioning is the more time consuming but is superior in that it (1) permits lens volume to be calculated and (2) enables the surface curvatures to be measured at the very center of the lens. Despite prior reports of success, we could not obtain satisfactory estimates of lens parameters in chicken eyes, using frozen sectioning. When tissue was chemically fixed before sectioning, we obtained insufficient contrast between the aqueous humor and the anterior surface of the crystalline lens to permit automated detection of the lens/aqueous boundary.When unfixed tissue was examined, we found that swelling of the lens occurred, invalidating the results obtained (ironically, however, image contrast between the lens and the surrounding tissue was excellent).

MRI has the potential to overcome many of the disadvantages of the techniques mentioned above. It enables all of the relevant lens parameters to be measured (indeed, it can even be used to determine the refractive index at any point in the crystalline lens [14]). Like frozen sectioning, MRI has the capacity to provide true 3D representations of the lens. Only when such a 3D model of the whole lens surface has been obtained is it possible to calculate the thickness, equatorial diameter, and radii of curvature of the lens at its center and with reference to the optical axis of the eye.

Apart from the amount of time required for manual processing of images, e.g., mri3dX analysis, the main disadvantages of MRI are that (1) it requires the use of complex expensive equipment, which usually means that access incurs a high per hour cost to the end user and (2) the time taken to acquire images is dependent on both the desired level of resolution and the 3D size of the structure being scanned. These latter two disadvantages are intimately related because the longer scan times necessary to scan small samples at high resolution obviously lead to higher scanning costs. We sought to strike a balance between image resolution and scanning costs by choosing to scan multiple (specifically, 16) eyes simultaneously and to scan for a long period (16 h). Interestingly, due to the nature of MRI and the 2×2×4 array system of arrangement of eyes used in the present study, this approach yielded a fourfold increase in resolution compared to scanning a single eye for 1 h.

However, our experience shows that it is not currently possible to obtain high-resolution 3D representations of small eyes or crystalline lenses using MRI (certainly not in the large numbers of animals required for a gene mapping study). To obtain sufficient image contrast to allow the dimensions of the chicken crystalline lens to be measured, our work shows that even with a new top-of-the-range MRI scanner, a scanning time of 4 h is required to provide an isotropic image resolution of approximately 120 μm. Thus, for an in vivo scan, it would be necessary to keep a young chicken anesthetized and absolutely motionless for 4 h, which is not feasible (and to carry out this feat on the hundreds of chickens required for a gene mapping study would have been prohibitively expensive). Where researchers have succeeded previously in obtaining high-resolution MRI scans of animal eyes, this has been done with high resolution in only two dimensions and poor resolution (e.g., 0.5–1.0 mm) in the third [15,16]. This “thick slice” approach is appealing because the MRI signal is integrated across the depth of the slice, producing good image contrast, but it leads to an averaged blurred representation of the eye’s structure. With the knowledge that in vivo MRI scanning could not provide high-resolution 3D images of the crystalline lens, we chose to scan ex vivo eyes so that long scan times could be used. Unfortunately, such ex vivo work requires the use of tissue fixation and, as discussed below, we found this to introduce dramatic alterations in lens shape.

### Kidney-shaped lenses

**Figure 6:**
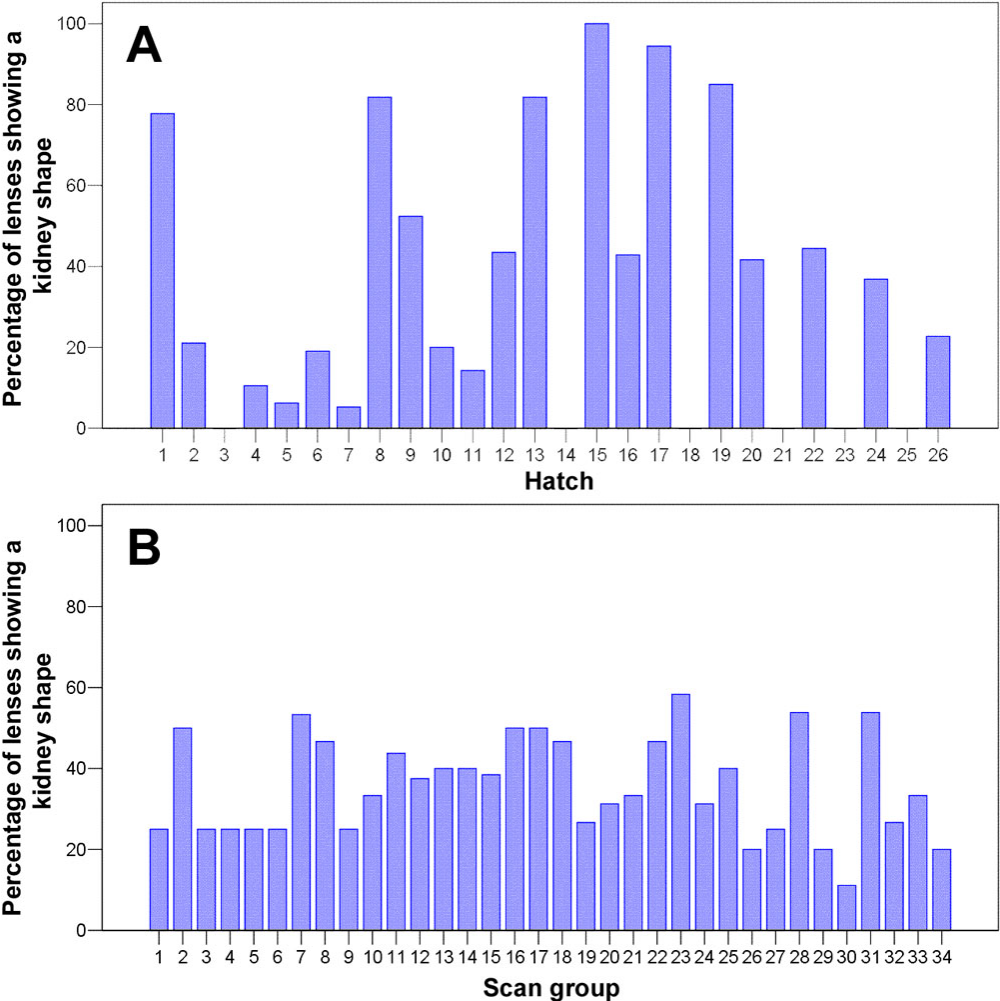
Presence/absence of a kidney-shaped appearance as a function of hatch and scan group. Panel **A** shows the proportion of kidney-shaped lenses as a function of hatch (i.e., batches of chickens hatched and phenotyped together). Note that for some hatches (e.g., hatches 3, 14, 18, 21, 23, and 25) none of the lenses had a kidney shape, yet for other hatches (e.g., hatches 15 and 17) kidney-shaped lenses were the norm. Panel **B** shows the proportion of kidney-shaped lenses as a function of scan group (i.e., groups of eyes that were MRI scanned at the same time). Note that the proportion of kidney-shaped lenses was approximately uniform across scan groups.

Our adoption of an ex vivo MRI scanning strategy necessitated the use of chemical fixation since unfixed lenses swell in buffered saline solution, thereby altering their dimensions within a matter of hours. A recent report by Augusteyn et al. [17] found that chemical fixation of the human crystalline lens with paraformaldehyde also caused its dimensions to change, although in this case to shrink rather than swell. This lens thinning after paraformaldehyde fixation was associated with a loss of water from the lens, particularly the lens cortex (why this water loss occurred was not clear [17]). We speculate that in the chicken lens, fixation-induced shrinkage is greatest in the central portion of the anterior lens cortex and that it is this localized shrinkage that leads to the warped kidney-shaped appearance noted in about one-third of the lenses examined in this study. In circumstantial support of this theory, we found that the proportion of kidney-shaped lenses was significantly associated with hatch (logistic regression, p<0.001; Figure 6A), i.e., that eyes fixed on the same day using the same batch of fixative solution had a greater than chance tendency to show a similar shape profile to one another. In contrast, the proportion of kidney-shaped lenses did not vary significantly as a function of whether or not eyes were MRI scanned together at the same time (logistic regression, p=0.80; Figure 6B). However, because hatch did not fully account for whether or not lenses appeared kidney shaped, other (unknown) factors must also be involved in causing the kidney-shape artifact.

In view of the significant influence of hatch on the prevalence of kidney-shaped lenses, we investigated whether restricting our analyses to those hatches with a low proportion of misshapen lenses would provide a less biased data set than our original analysis of lenses subjectively rated as nonkidney shaped. There were 8 hatches for which less than 10% of lenses were subjectively rated as kidney-shaped (these 8 hatches comprised of 146 lenses, only 2 of which were rated as kidney-shaped). For this group of lenses, the correlation between axial lens thickness measured by MRI and by ultrasound was r=0.42 (p<0.001). For the group of lenses from the hatches in which no lenses were rated as kidney shaped (111 lenses in 6 hatches), the correlation between axial lens thickness measured by MRI and lens thickness measured by ultrasound was similar (r=0.41, p<0.001). Coupled with scatter plots (not shown) of the relationship between lens thickness measured using the two methods, this suggested that restricting the analysis to specific hatches was unlikely to provide a less biased data set. For the hatches in which less than 10% of lenses were rated as kidney shaped, the correlations between lens parameters and other parameters are shown in Appendix 3. These correlations were similar to those observed for all eyes with lenses subjectively rated as nonkidney shaped (Appendix 2).

### Correlations in lens and eye size

The lens parameters measured by MRI were found to be subject to two distinct sources of error. First, a variable degree of fixation-induced shrinkage of the tissue was found to exert an overall downward bias and an increased variance in estimates of lens thickness (Figure 4A-D). In about one-third of the eyes, this resulted in an obvious change in lens shape that also compromised the accuracy of most of the other lens parameter measurements. Second, the scanning and image analysis procedures were found to introduce a relatively minor nonsystematic source of imprecision (Table 1), especially in measurements of equatorial lens diameter. The combination of these two sources of measurement imprecision made individual lens parameter estimates unreliable, which consequently made correlation coefficients between lens and eye size parameters also unreliable (because a random measurement error will act to lower the estimate of a correlation coefficient). Therefore, we chose to focus on whether a correlation coefficient was statistically significant rather than placing an emphasis on the actual magnitude of the correlation. (Note that some within-lens correlations, such as that between axial lens thickness and lens volume, were actually *higher* when lens thickness was measured by MRI rather than by ultrasound. We presume that this was due to shrinkage causing a correlated degree of change to both of these parameters, i.e., the effects of a *nonrandom* source of noise. For this reason, the magnitude of all within-lens correlations was regarded as particularly unreliable.)

Despite the reservations mentioned above, in this work we disclosed two interesting new findings regarding the co-regulation of the growth of the lens, the eye as a whole, and the rest of the body. First, we found that lens volume and lens equatorial diameter were both significantly related to eye and body size. This implies a common origin in the scaling of these structures, for instance due to a shared influence of specific genetic factors. In contrast, as we reported previously [6], axial lens thickness was only weakly related to eye and body size (as judged from ultrasound measurements) in these birds. There was also no evidence that the radii of curvature of the crystalline lens were related to eye and body size, but this lack of correlation could have been caused by the fixation artifact rather than representing the true physiologic situation. Together, our results suggest the novel theory that the volume and diameter of the lens might be under one system of control and the thickness (and possibly surface curvatures) of the lens under another. Animal studies have shown that visually guided refractive development typically has little effect on lens thickness [5]. Because changes to the depth of the vitreous chamber are the primary effecter of these visually guided responses, the low correlation between lens thickness and vitreous chamber depth in our population of chickens is consistent with these prior studies. In contrast, our finding of a modest but significant correlation between eye equatorial diameter and lens equatorial diameter is in keeping with the proposed link between these parameters that Zadnik, Mutti, and co-workers [1-4] speculate to be the cause of the reduction in lens power during childhood refractive development.

In conclusion, despite a serious source of measurement error as regards the dimensions of the crystalline lens due to the use of chemical fixation, we identified highly significant correlations between lens volume and eye/body size and between equatorial lens diameter and eye/body size in the chicken. These relationships contrast with the virtual independence noted previously between lens thickness and eye/body size, suggesting that different genetic or environmental factors might determine lens volume/diameter and lens thickness in normal chickens.

## Appendix 1. Spearman correlationsbetween axial lens thickness, measured using either MRI or ultrasound, and other lens, eye or body dimensions.

To access the table, click or select the words “Appendix 1.” This will initiate the download of a Word (.doc) file that contains the table.

## Appendix 2. Spearman correlations between lens dimensions measured using MRI and other lens, eye or body dimensions.

To access the table, click or select the words “Appendix 2.” This will initiate the download of a Word (.doc) file that contains the table.

## Appendix 3. Spearman correlations between lens dimensions measured using MRI and other lens, eye or body dimensions (data for eyes from batches with less than 10% kidney-shaped lenses).

To access the table, click or select the words “Appendix 3.” This will initiate the download of a Word (.doc) file that contains the table.
